# Prognostic Factors and Treatment Strategies for Elderly Patients with Malignant Meningioma: A SEER Population-Based Study

**DOI:** 10.3389/fonc.2022.913254

**Published:** 2022-05-13

**Authors:** Songshan Feng, Jing Li, Fan Fan, Zeyu Wang, Qian Zhang, Hao Zhang, Ziyu Dai, Xun Zhang, Peng Luo, Zaoqu Liu, Jian Zhang, Zhuoyi Liu, Quan Cheng

**Affiliations:** ^1^Department of Neurosurgery, Xiangya Hospital, Central South University, Changsha, Hunan, China; ^2^National Clinical Research Center for Geriatric Disorders, Xiangya Hospital, Central South University, Changsha, China; ^3^Xiangya Cancer Center, Xiangya Hospital, Central South University, Changsha, China; ^4^Key Laboratory of Molecular Radiation Oncology Hunan Province, Changsha, China; ^5^Department of Rehabilitation, the Second Xiangya Hospital, Central South University, Changsha, China; ^6^Department of Anesthesiology, Xiangya Hospital, Central South University, Changsha, China; ^7^Department of Oncology, Zhujiang Hospital, Southern Medical University, Guangzhou, China; ^8^Department of Interventional Radiology, The First Affiliated Hospital of Zhengzhou University, Zhengzhou, China; ^9^Department of Clinical Pharmacology, Xiangya Hospital, Central South University, Changsha, China

**Keywords:** malignant meningioma, elderly patient, treatment strategy, SEER, patient prognosis

## Abstract

**Objective:**

Malignant meningioma (MM) is a relatively rare disease with poor survival. Few studies had focused on MM in the elderly population. This study aims to explore the prognostic factors and optimal therapeutic strategy in elderly patients with MM.

**Methods:**

We took advantage of the Surveillance, Epidemiology, and End Results (SEER) database to include 275 adult patients with histologically confirmed MM between 2011 and 2018. The Kaplan–Meier curves were plotted by different covariates to reveal the survival probability. Univariate and multivariable Cox proportional hazard regression analyses were applied to identify prognostic factors for cancer-specific survival (CSS).

**Results:**

The multivariable analysis in the elderly group revealed that when compared with patients receiving gross total resection (GTR), patients receiving biopsy had significantly worse CSS (HR = 3.72; 95% CI: 1.35–10.21; P = 0.011), whereas patients receiving subtotal resection (STR) had nearly the same CSS (HR = 0.83; 95% CI: 0.37–1.86; P = 0.653). Meanwhile, postoperative radiotherapy (PORT) showed no significant association with CSS in the elderly patient group (HR = 0.94; 95% CI: 0.42–2.12; P = 0.888).

**Conclusion:**

Surgical resection is recommended for elderly patients with MM in the absence of surgical contraindications, but GTR does not present survival benefit in the elderly patients compared with STR. Additional large-scale clinical studies are needed to explore the survival benefit of PORT applied in patients with MM.

## Introduction

Meningioma is the most common primary neoplasm of the central nervous system, accounting for 38.3% of all brain tumors (1). According to the most recent report from the Central Brain Tumor Registry of the United States, malignant meningioma (MM) composes 1.04% of all meningiomas with an incidence of 0.09 per 100,000 people (1). There is evidence that age-specific incidence rates of meningiomas increase in both men and women, with a median age at diagnosis of 65 and 66 years old for malignant and non-malignant meningiomas, respectively (1, 2). As far as we know, most studies on MMs did not take the elderly (≥65 years old) as an independent patient group to describe (3). There were reports revealing that older age was associated with worse patient survival (4–7). Several studies suggested that craniotomy for resection of meningioma in the elderly patients carried higher risk of mortality and morbidity compared with younger patients (8, 9). Other studies reported that no significant difference was detected in the mortality rate after surgery for elderly versus non-elderly patients, but more elderly patients presented postoperative complications and neurological deterioration (10–12). At present, there is still a lack of consensus on the surgical outcome of elderly patients with MM, and the specific treatment strategies need to be further explored. Furthermore, it is expected that the average human life expectancy continues to increase and more elderly patients with MM will be diagnosed (10). Therefore, we conducted this study aiming to explore the prognostic factors and figure out the optimal therapeutic strategy, especially in elderly patients with MM.

## Materials and Methods

### Study Population

**Figure 1:**
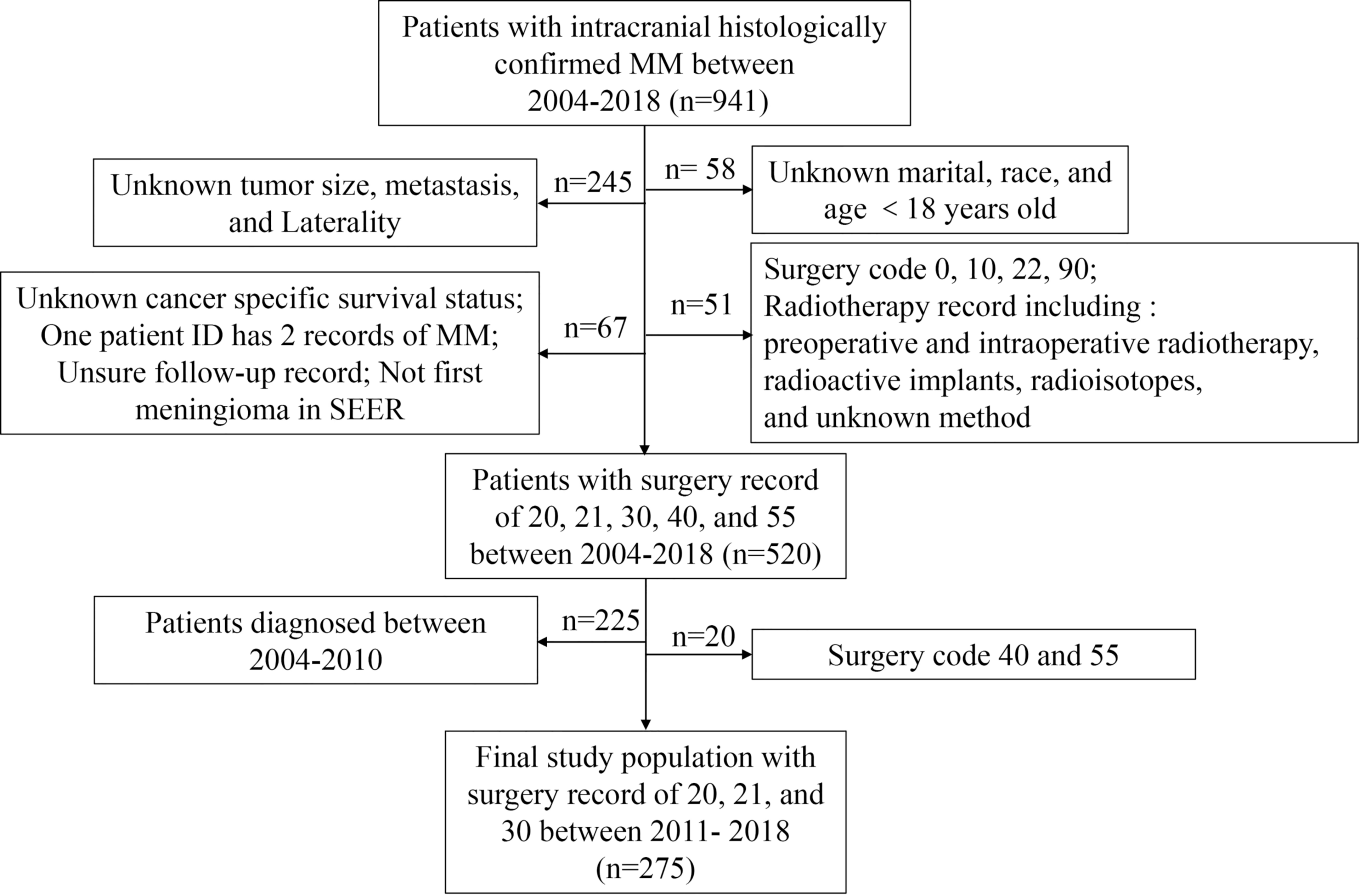
Flow chart of patient selection criteria with *de novo* MM between 2011 and 2018.

Given the low incidence of MM, we took advantage of the Surveillance, Epidemiology, and End Results (SEER) database and retrospectively analyzed 275 patients diagnosed with histologically confirmed MM between 2011 and 2018. The subgroup analysis for elderly and younger patients was performed with respect to extent of surgical resection (EOR), postoperative radiotherapy (PORT), and their influence on long-term patient survival. All records of intracranial MM with positive histology between 2004 and 2018 were initially extracted from the SEER database, which provides patient demographics, tumor characteristics, treatment methods, and survival status with de-identified records. WHO grade 3 meningioma was defined as MM, which included the ICD-O-3 histology and behavior records of 9530/3 (Meningioma, malignant), 9531/3 (Meningiothelial meningioma, malignant), 9532/3 (Fibrous meningioma, malignant), 9534/3 (Angiomatous meningioma, malignant), 9535/3 (Hemangioblastic meningioma, malignant), 9537/3 (Transitional meningioma, malignant), 9538/3 (Papillary meningioma), and 9539/3 (Meningeal sarcomatosis) according to existing studies (13, 14). Patients with unknown information of marital status, race, tumor size, laterality, cancer-specific survival (CSS) status, and age<18 years old were excluded. Patients with recurrent MM were also excluded, which had at least one prior record of WHO grade 1 or WHO grade 2 meningioma in the SEER database. The detailed protocol was provided by the SEER*Stat tutorial naming “case listing exercise 1b: view patient histories” (https://seer.cancer.gov/seerstat/tutorials/case-listings.html). Surgery code 0 (no surgery of primary site; autopsy only), code 10 (no specimen sent to pathology), code 22 (resection of tumor of spinal cord or nerve), and code 90 (surgery, not otherwise specified) were excluded. In addition, the small part of patients treated with radiotherapy prior to surgery, intraoperative radiotherapy, radioactive implants, radioisotopes, and unknown method were excluded. **Supplementary Table 1** showed that the records of surgery code changed significantly since 2011, which revealed the advancement in surgical techniques. To provide the most up-dated evidence, the patient diagnosed before 2011 were excluded, and little parts of patients with surgery code 40 (partial resection of lobe of brain) and 55 (gross total resection of lobe of brain) were also excluded (n = 20). The final study population included 275 adult patients diagnosed between 2011 and 2018 recorded as surgery code 20 (local excision, biopsy), 21 [subtotal resection (STR) of tumor], and 30 [radical, total, gross resection of tumor (GTR)] (**Figure 1**).

### Covariates Included

The following demographic information was included for analysis: age group (<65 and ≥65 years), gender (male and female), race (other, black, and white), and marital status (single, divorced, married, and widowed). The following tumor characteristics were analyzed: tumor site (cerebral meninges and other), laterality (unilateral, bilateral, and midline), histology (9530/3 and other), tumor size (≥4.9 and <4.9 cm, the best cutoff was defined by x-tile software) (15), and other tumor(s) (before MM, and after MM, defined by the record of “sequence number” in SEER*Stata). EOR includes code 20 (biopsy), code 21 (STR), and code 30 (GTR). Concerning adjuvant therapy, PORT

(no/unknown and beam radiation), and chemotherapy (no/unknown and yes) were included for analysis. CSS was defined as the event of interest in this study.

### Statistical Methods

The distribution of the baseline characteristics between different age groups was compared by the chi-squared test (categorical variables with all cell counts>5) or the Fisher’s exact test (categorical variables with cell counts ≤5). The Kaplan–Meier curves in the entire cohort were plotted by all covariates to reveal the CSS probability of different groups, which were compared by log-rank test. Univariate and multivariable Cox proportional hazard regression analyses were applied to identify prognostic factors from all covariates for CSS. The baseline characteristics between groups receiving different EOR were compared by the chi-squared test, Fisher’s exact test, or one-way ANOVA test (continuous variable). The Kaplan–Meier curves by EOR and PORT were plotted in elderly and younger patient group. Furthermore, univariate and multivariable Cox proportional hazard regression analyses were also applied to assess the survival benefits provided by EOR and PORT for younger and elderly patients, respectively. P < 0.05 was considered statistically significant. All statistical analyses were performed in R version 3.5.1 (http://www.r-project.org/).

## Results

### Baseline Characteristics

The median age was 62 years old, and median survival time was 28 months. At the time of data collected, 183 cases were alive, 56 cases died of MM, and 36 cases died of other causes. The 1-, 2-, and 5-year CSS rates were 88.5%, 80.7%, and 52.1%, respectively. The baseline characteristics were compared between age groups in **Table 1**. The marital status showed a significant difference, whereas race and gender showed no difference between age groups. The majority of patients had tumor larger than 4.9 cm, tumor located in cerebral meninges, unilateral tumor, tumor with histology 9530/3, tumor without metastasis, and no other tumor(s). Tumor characteristics including tumor size, site, laterality, histology, metastasis, and other tumor(s) showed no significant difference between different age groups (P > 0.05). Concerning treatment methods, the results revealed that the GTR rate was 52.4% in the entire cohort, 51.3% in the younger group, and 53.7% in the elderly group. Compared with patients in the younger group, more patients received biopsy only and fewer patients received STR in the elderly group. A total of 149 patients received PORT and 12 patients received postoperative chemotherapy, which showed no significant difference between age groups.

**Table 1:** Patient demographics, tumor characteristics, and treatment options of 275 patients with MM from 2011 to 2018 in different age groups.

	Overall [n (%)]	<65 years [n (%)]	≥65 years [n (%)]	P-value
No.	275 (100)	152 (100)	123 (100)	
Gender				0.052
Male	133 (48.4)	65 (42.8)	68 (55.3)	
Female	142 (51.6)	87 (57.2)	55 (44.7)	
Race				0.85
Other	33 (12.0)	17 (11.2)	16 (13.0)	
Black	43(15.6)	23 (15.1)	20 (16.3)	
White	199 (72.4)	112 (73.7)	87 (70.7)	
Marital				<0.001^†^
Single	64 (23.3)	48 (31.6)	16 (13.0)	
Divorced	29 (10.5)	12 (7.9)	17 (13.8)	
Married	159 (57.8)	86 (56.6)	73 (59.3)	
Widowed	23 (8.4)	6 (3.9)	17 (13.8)	
Site				0.506
Meninges	267 (97.1)	149 (98.0)	118 (95.9)	
Other	8 (2.9)	3 (2.0)	5 (4.1)	
Laterality				0.182
Unilateral	253 (92.0)	136 (89.5)	117 (95.1)	
Bilateral	2 (0.7)	1 (0.7)	1 (0.8)	
Midline	20 (7.3)	15 (9.9)	5 (4.1)	
Histology				0.577
9530/3	216 (78.5)	117 (77.0)	99 (80.5)	
Other	59 (21.5)	35 (23.0)	24 (19.5)	
Other tumors				0.068
One primary	203 (73.8)	120 (78.9)	83 (67.5)	
Before MM	51 (18.5)	21 (13.8)	30 (24.4)	
After MM	21 (7.6)	11 (7.2)	10 (8.1)	
Size				0.998
>4.9cm	133 (48.4)	73 (48.0)	60 (48.8)	
≤4.9cm	142 (51.6)	79 (52.0)	63 (51.2)	
Metastasis				0.627
No	268 (97.5)	147 (96.7)	121 (98.4)	
Yes	7 (2.5)	5 (3.3)	2 (1.6)	
Surgery code				0.04^†^
GTR	144 (52.4)	78 (51.3)	66 (53.7)	
Biopsy	38 (13.8)	15 (9.9)	23 (18.7)	
STR	93 (33.8)	59 (38.8)	34 (27.6)	
Chemotherapy				0.607
Yes	12 (4.4)	8 (5.3)	4 (3.3)	
No/Unknown	263 (95.6)	144 (94.7)	119 (96.7)	
PORT				0.237
Beam radiation	149 (54.2)	77 (50.7)	72 (58.5)	
No/Unknown	126 (45.8)	75 (49.3)	51 (41.5)	

^†^P < 0.05, statistically significant.

EOR, extent of surgery; GTR, gross total resection; STR, subtotal resection; PORT, postoperative radiotherapy; MM, malignant meningioma.

### Prognostic Factors of CSS in the Entire Cohort

**Figure 2:**
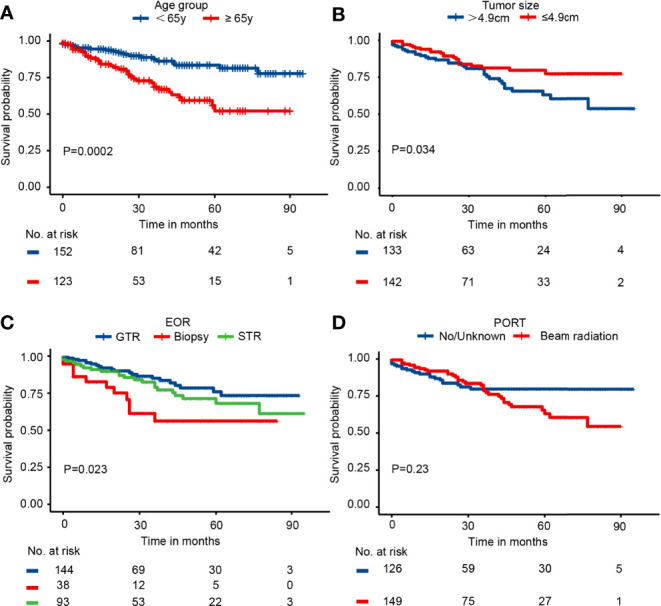
The Kaplan–Meier curves by **(A)** age group, **(B)** tumor size, **(C)** EOR, and **(D)** PORT in the entire cohort.

Kaplan–Meier curves indicated that patients in the elderly group, with tumor size>4.9 cm, receiving biopsy only, and receiving chemotherapy had significantly worse survival probability. In addition, the Kaplan–Meier curves by PORT, gender, race, marital status, histology, tumor site, laterality, metastasis, and other tumor(s) showed no significant difference (**Figure 2** and **Supplementary Figure 1**). The results of univariate analysis revealed that patients in elderly group (HR = 2.73; 95% CI: 1.57–4.74; P = 3.56e−4), with tumor size>4.9 cm (HR = 1.77; 95% CI: 1.04–3.04; P = 0.036), receiving biopsy only (HR = 2.62; 95% CI: 1.29–5.31; P = 0.007), and receiving chemotherapy (HR = 3.69; 95% CI: 1.75–7.81; P = 6.3e−4) presented significantly worse CSS. PORT, gender, race, marital status, histology, tumor site, laterality, metastasis, and other tumor(s) were not significantly associated with CSS (P > 0.05) (**Table 2** and **Supplementary Table 2**). Consistently, the results of the multivariable analysis revealed that patients in the elderly group (HR = 3.41; 95% CI: 1.86–6.23; P = 6.81e−5), with tumor size>4.9 cm (HR = 1.78; 95% CI: 1.01–3.16; P = 0.048), receiving biopsy only (HR = 3.03; 95% CI: 1.43–6.44; P = 0.004), and receiving chemotherapy (HR = 4.19; 95% CI: 1.77–9.90; P = 0.001) showed significant worse CSS. Meanwhile, PORT, gender, race, marital status, histology, tumor site, laterality, metastasis, and other tumor(s) were not significantly associated with CSS (P > 0.5) (**Table 2** and **Supplementary Table 2**).

**Table 2:** Results of univariate and multivariable Cox proportional regression analysis of age group, tumor size, EOR, and PORT in the entire study population.

	Univariate Analysis		Multivariable Analysis	
	HR (95% CI)	P-value	HR (95% CI)	P-value
Age				
<65 years	1 [Reference]		1 [Reference]	
≥65 years	2.73 (1.57–4.74)	3.56 × 10^−4†^	3.41 (1.86–6.23)	6.81 × 10^−5†^
Size				
≤4.9cm	1 [Reference]		1 [Reference]	
>4.9cm	1.77 (1.04–3.04)	0.036^†^	1.78 (1.01–3.16)	0.048^†^
Extent of resection				
GTR	1 [Reference]		1 [Reference]	
Biopsy	2.62 (1.29–5.31)	0.007^†^	3.03 (1.43–6.44)	0.004^†^
STR	1.40 (0.77–2.53)	0.262	1.23 (0.67–2.27)	0.497
PORT				
No/Unknown	1 [Reference]		1 [Reference]	
Beam radiation	1.29 (0.81–2.38)	0.235	0.81 (0.44–1.49)	0.503

^†^P < 0.05, statistically significant.

EOR, extent of surgery; GTR, gross total resection; STR, subtotal resection; PORT, postoperative radiotherapy.

### The Survival Benefits of EOR and PORT in Subgroups

**Figure 3:**
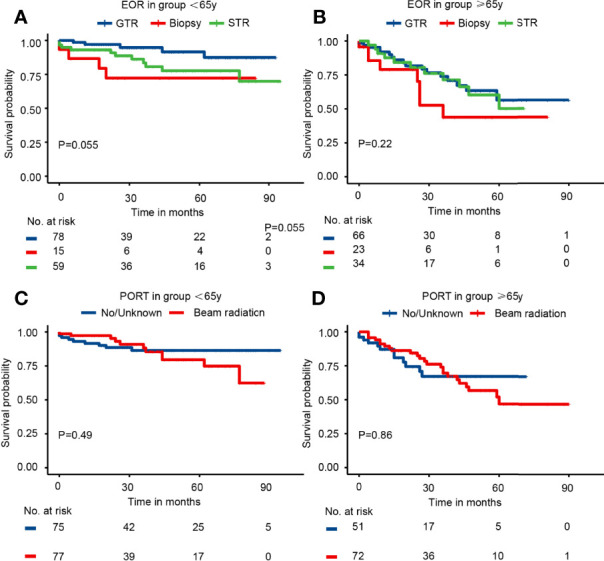
The Kaplan–Meier curves by EOR and PORT in different age groups. **(A)** EOR in group <65 years. **(B)** EOR in group ≥65 years. **(C)** PORT in group <65 years. **(D)** PORT in group ≥65 years.

The subgroup analysis of elderly and younger patients was conducted to assess the survival benefits of EOR and PORT. First, the Kaplan–Meier curves in the younger group indicated that patients receiving biopsy presented the worst survival probability, and patients receiving GTR had a slightly better survival probability than that receiving STR (P = 0.055). The Kaplan–Meier curves in the elderly group showed that the survival probability of patients receiving different EOR had no significant difference (P = 0.22). The Kaplan–Meier curves in both age groups suggested that PORT did not affect survival probability (**Figure 3**). The univariate analysis in the younger group showed that when compared with patients receiving GTR, patients receiving biopsy had significantly worse CSS (HR = 4.23; 95% CI: 1.13–15.81; P = 0.032) and patients receiving STR had slightly worse CSS (HR = 2.66; 95% CI: 0.93–7.69; P = 0.069). Meanwhile, the univariate analysis in the elderly group illustrated that when compared with patients receiving GTR, patients receiving biopsy had slightly worse CSS (HR = 2.07; 95% CI: 0.89–4.82; P = 0.091), but patients receiving STR possessed nearly the same CSS (HR = 1.09; 95% CI: 0.51–2.35; P = 0.808). The results of univariate analysis revealed that PORT presented no significant association with CSS in both younger group and elderly group (**Table 3**). Consistently, the multivariable analysis in the younger group suggested that when compared with patients receiving GTR, patients receiving biopsy had significantly worse CSS (HR = 6.47; 95% CI: 1.42–29.44; P = 0.018) and patients receiving STR had slightly worse CSS (HR = 2.77; 95% CI: 0.81–9.48; P = 0.103). The multivariable analysis in the elderly group revealed that when compared with patients receiving GTR, patients receiving biopsy had significantly worse CSS (HR = 3.72; 95% CI: 1.35–10.21; P = 0.011) and patients receiving STR had nearly the same CSS (HR = 0.83; 95% CI: 0.37–1.86; P = 0.653). At the same time, the results of multivariable analysis illustrated that PORT showed no significant association with CSS in both younger group and elderly group (**Table 3**). The results of univariate and multivariable analyses of gender, race, marital, tumor size, histology, site, laterality, metastasis, other tumor(s), and chemotherapy in different age groups were presented in **Supplementary Table 3** (<65 years) and **Supplementary Table 4** (≥65 years), respectively.

**Table 3:** Results of univariate and multivariable Cox proportional regression analysis of EOR and PORT in different age groups.

Patient groups			Univariate Analysis		Multivariable Analysis	
			HR (95% CI)	P-value	HR (95% CI)	P-value
EOR	<65 years	GTR	1 [Reference]		1 [Reference]	
		Biopsy	4.23 (1.13–15.81)	0.032^†^	6.47 (1.42–29.44)	0.018^†^
		STR	2.66 (0.93–7.69)	0.069	2.77 (0.81–9.48)	0.103
	≥65 years	GTR	1 [Reference]		1 [Reference]	
		Biopsy	2.07 (0.89–4.82)	0.091	3.72 (1.35–10.21)	0.011^†^
		STR	1.09 (0.51–2.35)	0.808	0.83 (0.37–1.86)	0.653
PORT	<65 years	No/unknown	1 [Reference]		1 [Reference]	
		Beam radiation	1.36 (0.56–3.31)	0.493	2.29 (0.27–19.05)	0.442
	≥65 years	No/unknown	1 [Reference]		1 [Reference]	
		Beam radiation	1.06 (0.53–2.13)	0.865	0.94 (0.42–2.12)	0.888

^†^P < 0.05, statistically significant.

EOR, extent of surgery; GTR, gross total resection; STR, subtotal resection; PORT, postoperative radiotherapy.

The baseline characteristics of patients were compared between groups receiving different EOR in **Supplementary Table 5** (<65 years) and **Supplementary Table 6** (≥65 years), respectively. The results suggested that the patient demographics and tumor characteristics such as age, gender, tumor size, and tumor location presented no significant difference between elderly patients receiving different EOR.

## Discussion

Because of the prolongation of life expectancy, the treatment strategy of meningiomas in elderly patients has become a more and more important issue. Thus, we utilized the SEER database and retrospectively analyzed 275 patients diagnosed as MM with long-term outcome results, aiming to explore the prognostic factors and figure out the optimal therapeutic strategy for this specific population.

Elderly patients are more likely to be accompanied by other diseases, resulting in poor physical condition before surgery. Considering the risk of surgery and the corresponding surgical morbidity and mortality, conservative treatment may be a reasonable choice for elderly patients. However, it was reported that elderly patients who received conservative treatment had increased tumor-related mortality compared with patients who underwent surgical resection (16). Furthermore, both the univariate and multivariable analysis in our study suggested that biopsy was significantly associated with worse CSS in elderly patients. The European Association of Neuro-Oncology guidelines suggested surgical resection followed by PORT for the treatment of patients with MM (17). However, the specific surgical benefits and the choice of surgical patterns need to be further discussed.

There were studies reporting that meningioma surgery in elderly patients presented a higher risk of mortality and morbidity compared to intracranial tumor surgery in general (8, 18). Steinberger et al. revealed in their study that old age was an independent predictor of morbidity and mortality in patients undergoing craniotomy for resection of meningioma (9). Ferroli et al. reported in their retrospective cohort study that postoperative complications and surgical complexity could significantly influence the early outcome in elderly patients undergoing brain tumor surgery, and postoperative complications was the only factor with a strong correlation to postoperative worsening at the 3-month follow-up (19). In another study, the authors reported that no significant difference was discovered regarding the 30-day mortality rate for elderly versus non-elderly patients, whereas elderly patients had a significantly higher complication rate compared with non-elderly patients (10). Boviatsis et al. also revealed that the mortality rate between the elderly group and the younger group was not significant, but more elderly patients were discharged having deteriorated neurologically in comparison to their preoperative status (11). Hence, although there is dispute on whether the surgical resection would increase the mortality rate or not, it is generally recognized that the incidence of postoperative complications is higher in elderly patients.

Regarding the EOR and its influence on the long-term patient survival, several studies revealed that GTR was a favorable factor for patient survival in the general population (20, 21). Other studies indicated that GTR was not significantly associated with patient survival (22, 23). One particular study suggested that the overall survival of patients treated with near total resection was better than patients treated with GTR (24). Taking age into consideration, Brokinkel et al. reported that the progression-free interval of patients undergoing GTR was distinctly prolonged as compared with STR and emphasized the importance of achieving maximum safe resection in elderly patients (25). Another study also reported that the EOR had no influence on the functional outcome of elderly patients (26). However, D’Andrea et al. indicated that radical resection could increase postoperative morbidity in elderly patients (27). In another study, Chen et al. suggested that the aggressive resection of meningiomas in elderly patients could increase the morbidity and mortality, and survival with residual tumor was acceptable in this specific population (28). Our results revealed that GTR only improved CSS in younger patients compared with STR but did not present survival benefit in elderly patients. Therefore, we believe that surgeons should take into account the factors that the elderly are more prone to surgical complications when formulating surgical strategy for this special patient group, and a more balanced choice should be made in the pursuit of GTR and preservation of neurological function, so as to improve the postoperative functional status and survival of elderly patients.

Generally, PORT is recommended after tumor resection for the treatment of MM (17). There was supporting evidence revealing that PORT improved the survival of patients with MM (29, 30). Orton reported that PORT improved the overall survival of patients with MM undergoing both GTR and STR (4). However, another study illustrated that patients with MM did not benefit from PORT (20). For elderly patients with MM specifically, Zhou et al. and Achey et al. both suggested that PORT could not provide survival benefits after GTR (6, 31). The results of univariate analysis and multivariable analysis in our study showed that PORT exhibited no significant association with CSS in both younger group and elderly group. However, there may be a selection bias that those patients considered to possess a higher risk of recurrence or with more aggressive tumors are more likely to receive PORT. In addition, the information about PORT is not complete in the SEER database, which may affect the accuracy of the conclusion. We believe that additional large-scale clinical studies are needed to explore the survival benefit of PORT applied in patients with MM.

We are aware of the limitations of this study. The patients extracted from the SEER database may not represent the general patient cohort. For the elderly population, the concomitant disease before surgery, the complications, and functional status after surgery are important factors and may affect patient survival largely, which could not be obtained through the SEER database. Furthermore, the records of Simpson grades of resection and the exact radiotherapy information are also not available. Moreover, the insufficient number of patients may affect the analysis results and lead to selection bias.

## Conclusion

Surgical resection is recommended for elderly patients with MM in the absence of surgical contraindications, but GTR do not present survival benefit in the elderly patients compared with STR. Meanwhile, PORT exhibits no significant association with CSS in elderly group. Additional large-scale clinical studies are needed to explore the survival benefit of PORT applied in patients with MM. Despite several limitations, we believe that this study would help clinicians evaluate the prognosis of patients with MM and optimize treatment strategies for elderly patients specifically.

## Data Availability Statement

The original contributions presented in the study are included in the article/**Supplementary Material**. Further inquiries can be directed to the corresponding authors.

## Author Contributions

SF, ZYL, and QC made substantial contributions to the design of this study. SF, JL, FF, ZW, ZQL, and QC carried out the analysis and interpreted the data. SF, ZD, HZ, and JL made contributions to the drafting of the manuscript. ZD, QZ, PL, JZ, and XZ made contributions to the review of previous literature. SF, ZYL, and QC contributed substantially to the revision of the manuscript. All authors contributed to the article and approved the submitted version.

## Funding

This work was supported by the National Natural Science Foundation of China (Nos. 82102848, 82073893, and 81703622), China Postdoctoral Science Foundation (No. 2018M633002), Hunan Provincial Natural Science Foundation of China (Nos. 2018JJ3838 and 2020JJ8111), and Xiangya Hospital Central South University postdoctoral foundation.

## Conflict of Interest

The authors declare that the research was conducted in the absence of any commercial or financial relationships that could be construed as a potential conflict of interest.

## Publisher’s Note

All claims expressed in this article are solely those of the authors and do not necessarily represent those of their affiliated organizations, or those of the publisher, the editors and the reviewers. Any product that may be evaluated in this article, or claim that may be made by its manufacturer, is not guaranteed or endorsed by the publisher.
