# Supplementary material for: Prognostic Factors and Treatment Strategies for Elderly Patients with Malignant Meningioma: A SEER Population-Based Study
Source: Front Oncol. 2022 May 13;12:913254. doi: 10.3389/fonc.2022.913254 (PMC9136104; doi:10.3389/fonc.2022.913254)

|  | Overall | Code 30 | Code 20 | Code 21 | Code 40 | Code 55 | p |
| --- | --- | --- | --- | --- | --- | --- | --- |
| N (%) | 520(100) | 162(31.1) | 84(16.2) | 99(19.0) | 46(8.8) | 129(24.8) |  |
| Year |  |  |  |  |  |  | <0.001 |
| 2004 | 39(100) | 1(2.5) | 2(5.1) | 1(2.6) | 11(28.2) | 24(61.5) |  |
| 2005 | 36(100) | 4(11.1) | 3(8.3) | 0(0.0) | 7(19.4) | 22(61.1) |  |
| 2006 | 22(100) | 0(0.0) | 1(4.5) | 0(0.0) | 3(13.6) | 18(81.8) |  |
| 2007 | 39(100) | 3(7.7) | 7(17.9) | 0(0.0) | 8(20.5) | 21(53.8) |  |
| 2008 | 24(100) | 0(0.0) | 12(50) | 1(4.2) | 4(16.7) | 7(29.2) |  |
| 2009 | 28(100) | 2(7.1) | 10(35.7) | 1(3.6) | 7(25.0) | 8(28.6) |  |
| 2010 | 37(100) | 8(21.6) | 11(29.7) | 3(8.1) | 4(10.8) | 11(29.7) |  |
| 2011 | 36(100) | 17(47.2) | 3(8.3) | 12(33.3) | 1(2.8) | 3(8.3) |  |
| 2012 | 39(100) | 15(38.5) | 6(15.4) | 15(38.5) | 0(0) | 3(7.7) |  |
| 2013 | 43(100) | 21(48.8) | 7(16.3) | 12(27.9) | 0(0) | 3(7.0) |  |
| 2014 | 43(100) | 17(39.5) | 5(11.6) | 17(39.5) | 1(2.3) | 3(7.0) |  |
| 2015 | 32(100) | 13(40.6) | 7(21.9) | 11(34.4) | 0(0.0) | 1(3.2) |  |
| 2016 | 39(100) | 23(59.0) | 3(7.7) | 11(28.2) | 0(0.0) | 2(5.1) |  |
| 2017 | 40(100) | 23(57.5) | 4(10) | 12(30) | 0(0.0) | 1(2.5) |  |
| 2018 | 23(100) | 15(65.2) | 3(13.0) | 3(13.0) | 0(0.0) | 2(8.7) |  |
| Year era |  |  |  |  |  |  | <0.001 |
| 2004-2010 | 225(100) | 18(8.0) | 46(20.4) | 6(2.7) | 44(19.6) | 111(49.3) |  |
| 2011-2018 | 295(100) | 144(48.8) | 38(12.9) | 93(31.5) | 2(0.7) | 18(6.1) |  |

**Supplementary table 1. The distribution of surgery codes from 2004 -2018.**

**Supplementary Table 2.** **Results of univariate and multivariable Cox proportional regression analysis of other covariates in the entire study population.**

|  | Univariate analysis | | Multivariable analysis | |
| --- | --- | --- | --- | --- |
|  | HR (95%CI) | P-value | HR (95%CI) | P-value |
| Gender |  |  |  |  |
| Female | 1 [Reference] |  | 1 [Reference] |  |
| Male | 1.16 (0.69-1.97) | 0.569 | 0.81(0.45-1.47) | 0.495 |
| Race |  |  |  |  |
| Other | 1 [Reference] |  | 1 [Reference] |  |
| Black | 0.54(0.66-1.76) | 0.305 | 0.42(0.12-1.46) | 0.175 |
| White | 1.30 (0.55-3.04) | 0.551 | 0.05(0.43-2.56) | 0.911 |
| Marital status |  |  |  |  |
| Single | 1 [Reference] |  | 1 [Reference] |  |
| Divorced | 2.59(1.07-6.26) | 0.034^†^ | 2.29(0.89-5.93) | 0.087 |
| Married | 1.38(0.66-2.88) | 0.394 | 1.07(0.49-2.33) | 0.860 |
| Widowed | 0.90(0.24-3.31) | 0.868 | 0.47(0.12-1.79) | 0.266 |
| Histology |  |  |  |  |
| 9530/3 | 1 [Reference] |  | 1 [Reference] |  |
| Other | 0.63(0.31-1.28) | 0.199 | 0.67(0.32-1.39) | 0.278 |
| Site |  |  |  |  |
| Cerebral meninges | 1 [Reference] |  | 1 [Reference] |  |
| Other | / | / | / | / |
| Laterality |  |  |  |  |
| Unilateral | 1 [Reference] |  | 1 [Reference] |  |
| Bilateral | / | / | / | / |
| Midline | 0.48(0.12-1.98) | 0.310 | 0.45(0.10-1.98) | 0.289 |
| Metastasis |  |  |  |  |
| No | 1 [Reference] |  | 1 [Reference] |  |
| Yes | 0.72(0.10-5.19) | 0.743 | 0.52(0.06-4.28) | 0.543 |
| Other tumors |  |  |  |  |
| No other tumor | 1 [Reference] |  | 1 [Reference] |  |
| Before MM | 0.79(0.34-1.87) | 0.600 | 0.43(0.17-1.10) | 0.079 |
| After MM | 1.12(0.44-2.83) | 0.809 | 1.82(0.69-4.81) | 0.221 |
| Chemotherapy |  |  |  |  |
| No/Unknown | 1 [Reference] |  | 1 [Reference] |  |
| Yes | 3.69(1.75-7.81) | 6.3x10^-4†^ | 4.19 (1.77-9.90) | 0.001^†^ |

**Note:** ^†^P<0.05, statistically significant.

**Supplementary Table 3.** **Results of univariate and multivariable Cox proportional regression analysis of other covariates in the younger group.**

|  | Univariate analysis | | Multivariable analysis | |
| --- | --- | --- | --- | --- |
|  | HR (95%CI) | P-value | HR (95%CI) | P-value |
| Gender |  |  |  |  |
| Female | 1 [Reference] |  | 1 [Reference] |  |
| Male | 1.42(0.59-3.42) | 0.431 | 1.37(0.51-3.67) | 0.526 |
| Race |  |  |  |  |
| Other | 1 [Reference] |  | 1 [Reference] |  |
| Black | / | / | / | / |
| White | 3.35(0.45-25.04) | 0.239 | 1.74(0.20-14.87) | 0.610 |
| Marital status |  |  |  |  |
| Single | 1 [Reference] |  | 1 [Reference] |  |
| Divorced | 2.22(0.49-10.05) | 0.297 | 2.65(0.48-14.62) | 0.261 |
| Married | 1.44(0.46-4.47) | 0.531 | 0.68(0.18-2.56) | 0.569 |
| Widowed | 2.02(0.23-18.08) | 0.530 | 0.99(0.08-12.95) | 0.996 |
| Tumor size |  |  |  |  |
| ≤4.9cm | 1 [Reference] |  | 1 [Reference] |  |
| ＞4.9cm | 2.72(1.05-7.11) | 0.040^†^ | 2.82 (0.97-8.22) | 0.057 |
| Histology |  |  |  |  |
| 9530/3 | 1 [Reference] |  | 1 [Reference] |  |
| Other | 0.95(0.34-2.63) | 0.921 | 0.60(0.19-1.96) | 0.401 |
| Site |  |  |  |  |
| Cerebral meninges | 1 [Reference] |  | 1 [Reference] |  |
| Other | / | / | / | / |
| Laterality |  |  |  |  |
| Unilateral | 1 [Reference] |  | 1 [Reference] |  |
| Bilateral | / | / | / | / |
| Midline | 0.46(0.06-3.45) | 0.450 | 0.17(0.01-2.32) | 0.185 |
| Metastasis |  |  |  |  |
| No | 1 [Reference] |  | 1 [Reference] |  |
| Yes | 1.74(0.23-13.09) | 0.587 | 0.93(0.06-15.00) | 0.960 |
| Other tumors |  |  |  |  |
| No other tumor | 1 [Reference] |  | 1 [Reference] |  |
| Before MM | 0.55(0.07-4.24) | 0.570 | 0.93(0.10-8.71) | 0.952 |
| After MM | 2.21(0.63-7.66) | 0.209 | 6.45(1.48-27.94) | 0.013^†^ |
| Chemotherapy |  |  |  |  |
| No/Unknown | 1 [Reference] |  | 1 [Reference] |  |
| Yes | 6.27(2.28-17.29) | 3.8x10^-4†^ | 6.57(1.84-23.58) | 0.004^†^ |

**Note:** ^†^P<0.05, statistically significant.

**Supplementary Table 4. Results of univariate and multivariable Cox proportional regression analysis of other covariates in the elderly group.**

|  | Univariate analysis | | Multivariable analysis | |
| --- | --- | --- | --- | --- |
|  | HR (95%CI) | P-value | HR (95%CI) | P-value |
| Gender |  |  |  |  |
| Female | 1 [Reference] |  | 1 [Reference] |  |
| Male | 0.86(0.45-1.67) | 0.678 | 0.76(0.36-1.61) | 0.478 |
| Race |  |  |  |  |
| Other | 1 [Reference] |  | 1 [Reference] |  |
| Black | 0.61(0.17-2.12) | 0.437 | 0.65(0.16-2.68) | 0.551 |
| White | 0.84(0.32-2.19) | 0.716 | 0.97(0.34-2.81) | 0.959 |
| Marital status |  |  |  |  |
| Single | 1 [Reference] |  | 1 [Reference] |  |
| Divorced | 1.68(0.55-5.17) | 0.360 | 1.39(0.37-5.28) | 0.622 |
| Married | 0.87(0.32-2.32) | 0.788 | 0.82(0.27-2.48) | 0.725 |
| Widowed | 0.32(0.06-1.69) | 0.182 | 0.23(0.04-1.38) | 0.107 |
| Tumor size |  |  |  |  |
| ≤4.9cm | 1 [Reference] |  | 1 [Reference] |  |
| ＞4.9cm | 1.42(0.73-2.75) | 0.297 | 1.53 (0.71-3.28) | 0.274 |
| Histology |  |  |  |  |
| 9530/3 | 1 [Reference] |  | 1 [Reference] |  |
| Other | 0.50(0.18-1.42) | 0.195 | 0.50(0.17-1.48) | 0.211 |
| Site |  |  |  |  |
| Cerebral meninges | 1 [Reference] |  | 1 [Reference] |  |
| Other | / | / | / | / |
| Laterality |  |  |  |  |
| Unilateral | 1 [Reference] |  | 1 [Reference] |  |
| Bilateral | / | / | / | / |
| Midline | 1.07(0.15-7.89) | 0.944 | 0.70(0.09-5.71) | 0.741 |
| Metastasis |  |  |  |  |
| No | 1 [Reference] |  | 1 [Reference] |  |
| Yes | / | / | / | / |
| Other tumors |  |  |  |  |
| No other tumor | 1 [Reference] |  | 1 [Reference] |  |
| Before MM | 0.66(0.25-1.71) | 0.394 | 0.35(0.11-1.09) | 0.069 |
| After MM | 0.57(0.13-2.40) | 0.448 | 1.14(0.24-5.29) | 0.865 |
| Chemotherapy |  |  |  |  |
| No/Unknown | 1 [Reference] |  | 1 [Reference] |  |
| Yes | 2.76(0.84-9.06) | 0.094^†^ | 3.12(0.79-12.38) | 0.105 |

**Note:** ^†^P<0.05, statistically significant.

**Supplementary Table 5. The baseline characteristics of younger patients between groups receiving different EOR.**

|  | GTR | Biopsy | STR | p-value |
| --- | --- | --- | --- | --- |
| No. | 78 | 15 | 59 |  |
| Age (mean (SD)) | 50.56 (11.53) (11.53) | 53.27 (7.74) | 49.14 (13.06) | 0.464 |
| Gender |  |  |  | 0.303 |
| Male | 37 (47.4) | 4 (26.7) | 24 (40.7) |  |
| Female | 41(52.6) | 11(73.3) | 35(59.3) |  |
| Race |  |  |  | 0.379 |
| Other | 7 (9.0) | 2 (13.3) | 8 (13.6) |  |
| Black | 16 (20.5) | 2 (13.3) | 5 (8.5) |  |
| White | 55 (70.5) | 11 (73.3) | 46 (78.0) |  |
| Marital |  |  |  | 0.811 |
| Single | 25 (32.1) | 6 (40.0) | 17 (28.8) |  |
| Divorced | 8 (10.3) | 0 (0.0) | 4 (6.8) |  |
| Married | 42 (53.8) | 8 (53.3) | 36 (61.0) |  |
| Widowed | 3 (3.8) | 1 (6.7) | 2 (3.4) |  |
| Site |  |  |  | 0.234 |
| Meninges | 75(96.2) | 15 (100.0) | 59 (100.0) |  |
| Other | 3 (3.8) | 0 (0.0) | 0 (0.0) |  |
| Laterality |  |  |  | 0.58 |
| Unilateral | 70 (89.7) | 12 (80.0) | 54 (91.5) |  |
| Bilateral | 1 (1.3) | 0 (0.0) | 0 (0.0) |  |
| Midline | 7 (9.0) | 3 (20.0) | 5 (8.5) |  |
| Histology |  |  |  | 0.11 |
| 9530/3 | 62(79.5) | 14(93.3) | 41(69.5) |  |
| Other | 16 (20.5) | 1 (6.7) | 18 (30.5) |  |
| Other tumors |  |  |  | 0.238 |
| One primary | 56 (71.8) | 12 (80.0) | 52 (88.1) |  |
| Before MM | 14 (17.9) | 2 (13.3) | 5 (8.5) |  |
| After MM | 8 (10.3) | 1 (6.7) | 2 (3.4) |  |
| Size |  |  |  | 0.447 |
| ＞4.9cm | 35 (44.9) | 6 (40.0) | 32 (54.2) |  |
| ≤4.9cm | 43(55.1) | 9(60.0) | 27(45.8) |  |
| Metastasis |  |  |  | 0.346 |
| No | 77(98.7) | 14(93.3) | 56(94.9) |  |
| Yes | 1 (1.3) | 1 (6.7) | 3 (5.1) |  |
| Chemotherapy |  |  |  | 0.185 |
| Yes | 2 (2.6) | 2 (13.3) | 4 (6.8) |  |
| No/Unknown | 76(97.4) | 13(86.7) | 55(93.2) |  |
| PORT |  |  |  | 0.042^†^ |
| Beam radiation | 43 (55.1) | 3 (20.0) | 31 (52.5) |  |
| No/Unknown | 35(44.9) | 12(80.0) | 28(47.5) |  |

**Abbreviation**: GTR: Gross total resection; STR: Subtotal Resection; PORT: Post Operative Radiotherapy; MM: Malignant Meningioma; **Note:** ^†^P<0.05, statistically significant.

**Supplementary Table 6. The baseline characteristics of elderly patients between groups receiving different EOR.**

|  | GTR | Biopsy | STR | p-value |
| --- | --- | --- | --- | --- |
| No. | 66 | 23 | 34 |  |
| Age (mean (SD)) | 74.39 (7.67) | 71.39 (5.43) (5.43) | 72.09 (6.55) | 0.12 |
| Gender |  |  |  | 0.447 |
| Male | 38 (57.6) | 10 (43.5) | 20 (58.8) |  |
| Female | 28 (42.4) | 13 (56.5) | 14 (41.2) |  |
| Race |  |  |  | 0.095 |
| Other | 12 (18.2) | 3 (13.0) | 1 (2.9) |  |
| Black | 11 (16.7) | 1 (4.3) | 8 (23.5) |  |
| White | 43 (65.2) | 19 (82.6) | 25 (73.5) |  |
| Marital |  |  |  | 0.574 |
| Single | 9 (13.6) | 3 (13.0) | 4 (11.8) |  |
| Divorced | 5 (7.6) | 5 (21.7) | 7 (20.6) |  |
| Married | 42 (63.6) | 12 (52.2) | 19 (55.9) |  |
| Widowed | 10 (15.2) | 3 (13.0) | 4 (11.8) |  |
| Site |  |  |  | 0.926 |
| Meninges | 63 (95.5) | 22 (95.7) | 33 (97.1) |  |
| Other | 3 (4.5) | 1 (4.3) | 1 (2.9) |  |
| Laterality |  |  |  | 0.301 |
| Unilateral | 64 (97.0) | 21 (91.3) | 32 (94.1) |  |
| Bilateral | 0 (0.0) | 1 (4.3) | 0 (0.0) |  |
| Midline | 2 (3.0) | 1 (4.3) | 2 (5.9) |  |
| Histology |  |  |  | 0.707 |
| 9530/3 | 52 (78.8) | 18 (98.3) | 29 (85.3) |  |
| Other | 14 (21.2) | 5 (1.7) | 5 (14.7) |  |
| Other tumors |  |  |  | 0.473 |
| One primary | 46 (69.7) | 13 (56.5) | 24 (70.6) |  |
| Before MM | 16 (24.2) | 6 (26.1) | 8 (23.5) |  |
| After MM | 4 (6.1) | 4 (17.4) | 2 (5.9) |  |
| Size |  |  |  | 0.256 |
| ＞4.9cm | 36 (54.5) | 8 (34.8) | 16 (47.1) |  |
| ≤4.9cm | 30 (45.5) | 15 (65.2) | 18 (52.9) |  |
| Metastasis |  |  |  | 0.442 |
| No | 65 (98.5) | 22 (95.7) | 34 (100) |  |
| Yes | 1 (1.5) | 1 (4.3) | 0 (0.0) |  |
| Chemotherapy |  |  |  | 0.093 |
| Yes | 1 (1.5) | 0 (0.0) | 3 (8.8) |  |
| No/Unknown | 65 (98.5) | 23 (100) | 31 (91.2) |  |
| PORT |  |  |  | 0.425 |
| Beam radiation | 37 (56.1) | 12 (52.2) | 23 (67.6) |  |
| No/Unknown | 29 (43.9) | 11 (47.8) | 11 (32.4) |  |

**Abbreviation**: GTR: Gross total resection; STR: Subtotal Resection; PORT: Post Operative Radiotherapy; MM: Malignant Meningioma

**Supplementary Figure 1.** Kaplan-Meier curves by different covariates in the entire cohort. **(A)** Sex. **(B)** Race. **(C)** Marital status. **(D)** Histology. (**E**) Site. (**F**) Laterality. (**G**) Metastasis. (**H**) Other tumor(s). **(I)** Chemotherapy.


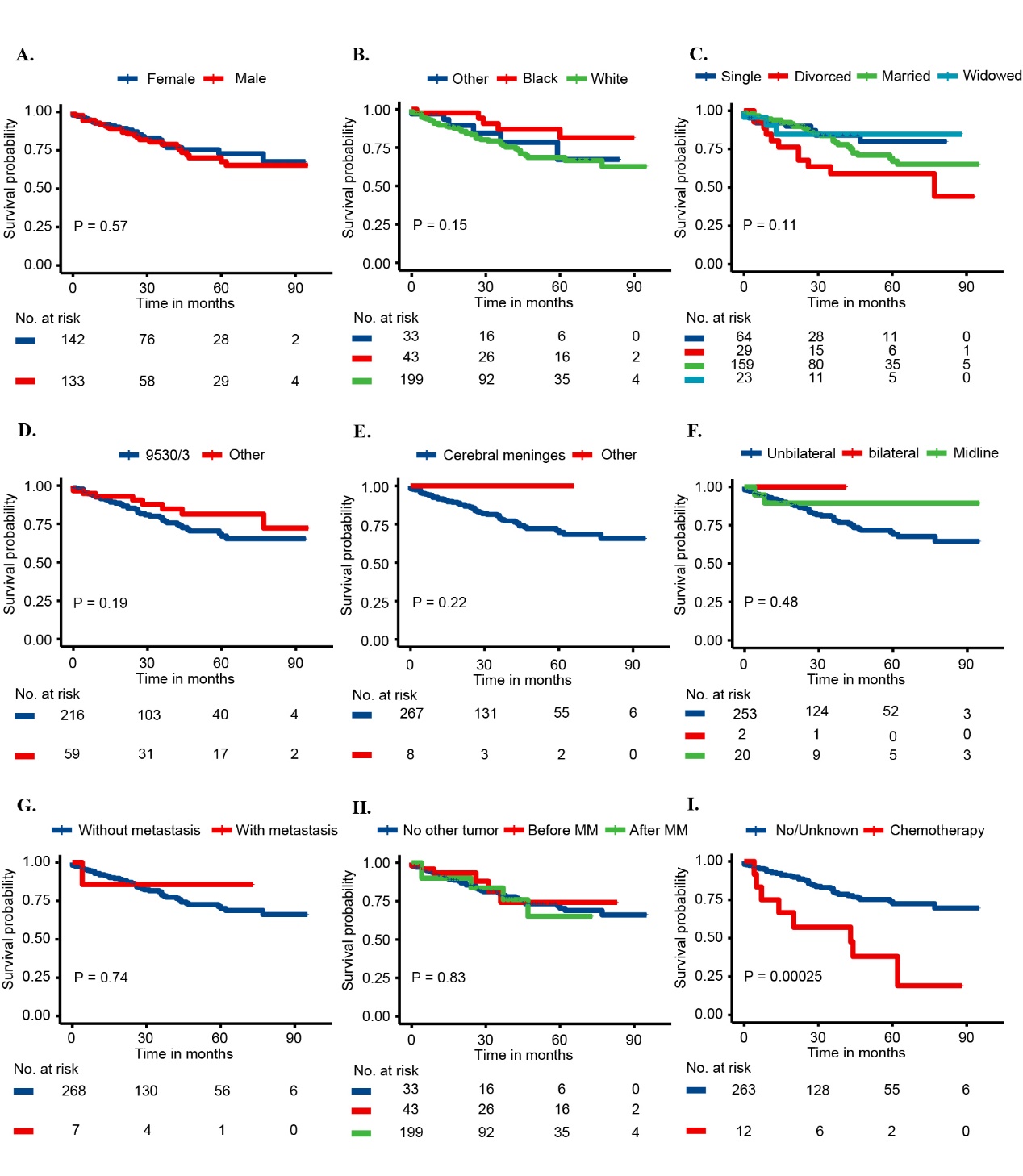

Supplement: Supplementary file 1 [file DataSheet_1.docx]
